# Molecular mapping of genomic regions and identification of possible candidate genes associated with gynoecious sex expression in bitter gourd

**DOI:** 10.3389/fpls.2023.1071648

**Published:** 2023-03-02

**Authors:** Vinay N. D., Hideo Matsumura, Anilabha Das Munshi, Ranjith Kumar Ellur, Viswanathan Chinnusamy, Ankita Singh, Mir Asif Iquebal, Sarika Jaiswal, Gograj Singh Jat, Ipsita Panigrahi, Ambika Baladev Gaikwad, A. R. Rao, Shyam Sundar Dey, Tusar Kanti Behera

**Affiliations:** ^1^ Division of Vegetable Science, ICAR-Indian Agricultural Research Institute, New Delhi, India; ^2^ Gene Research Centre, Shinshu University, Ueda, Nagano, Japan; ^3^ Division of Genetics, ICAR-Indian Agricultural Research Institute, New Delhi, India; ^4^ Division of Plant Physiology, ICAR-Indian Agricultural Research Institute, New Delhi, India; ^5^ Centre for Agricultural Bioinformatics, ICAR-Indian Agricultural Statistics Research Institute, New Delhi, India; ^6^ Division of Genomic Resources, ICAR-National Bureau of Plant Genetic Resources, New Delhi, India; ^7^ ICAR-Indian Institute of Vegetable Research, Varanasi, Uttar Pradesh, India

**Keywords:** bitter gourd (*Momordica charantia*), sex expression, gynoecious, inheritance, QTL-seq, candidate genes

## Abstract

Bitter gourd is an important vegetable crop grown throughout the tropics mainly because of its high nutritional value. Sex expression and identification of gynoecious trait in cucurbitaceous vegetable crops has facilitated the hybrid breeding programme in a great way to improve productivity. In bitter gourd, gynoecious sex expression is poorly reported and detailed molecular pathways involve yet to be studied. The present experiment was conducted to study the inheritance, identify the genomic regions associated with gynoecious sex expression and to reveal possible candidate genes through QTL-seq. Segregation for the gynoecious and monoecious sex forms in the F_2_ progenies indicated single recessive gene controlling gynoecious sex expression in the genotype, PVGy-201. Gynoecious parent, PVGy-201, Monoecious parent, Pusa Do Mausami (PDM), and two contrasting bulks were constituted for deep-sequencing. A total of 10.56, 23.11, 15.07, and 19.38 Gb of clean reads from PVGy-201, PDM, gynoecious bulk and monoecious bulks were generated. Based on the ΔSNP index, 1.31 Mb regions on the chromosome 1 was identified to be associated with gynoecious sex expression in bitter gourd. In the QTL region 293,467 PVGy-201 unique variants, including SNPs and indels, were identified. In the identified QTL region, a total of 1019 homozygous variants were identified between PVGy1 and PDM genomes and 71 among them were non-synonymous variants (SNPS and INDELs), out of which 11 variants (7 INDELs, 4 SNPs) were classified as high impact variants with frame shift/stop gain effect. In total twelve genes associated with male and female gametophyte development were identified in the QTL-region. Ethylene-responsive transcription factor 12, Auxin response factor 6, Copper-transporting ATPase RAN1, CBL-interacting serine/threonine-protein kinase 23, ABC transporter C family member 2, DEAD-box ATP-dependent RNA helicase 1 isoform X2, Polygalacturonase QRT3-like isoform X2, Protein CHROMATIN REMODELING 4 were identified with possible role in gynoecious sex expression. Promoter region variation in 8 among the 12 genes indicated their role in determining gynoecious sex expression in bitter gourd genotype, DBGy-1. The findings in the study provides insight about sex expression in bitter gourd and will facilitate fine mapping and more precise identification of candidate genes through their functional validation.

## Introduction

*Momordica charantia* L. (2n=22), often known as bitter gourd or bitter melon, is a prominent vegetable cum medicinal plant grown widely in India, China, Malaysia, Africa, and South America (Heiser, 1979). Bitter gourd is rich source of ascorbic acid and iron, and is renowned for its anti-diabetic, anti-carcinogenic, and anti-HIV properties (Behera, 2004; Behera et al., 2010). Indian bitter gourd germplasm exhibits wide phenotypic diversity for growth habit, maturity, fruit shape, size, colour, surface texture and sex expression (Robinson and Decker-Walters, 1999). To exploit genetic variation for crop improvement, it is essential to understand genetic and molecular basis of the traits under consideration.

In flowering plants, sex determination is a key developmental process of great biological significance (Kater et al., 2001). The family *Cucurbitaceae*, is regarded as model to study the physiological and molecular mechanisms of sex determination (Bhowmick and Jha, 2015). Monoecy, bearing separate male and female unisexual flowers on the same plant, is predominant sex form in Cucurbitaceae than the more typical bisexual flowers in higher plants (Behera, 2004). Genetic and molecular basis of sex determination is well documented in two major *Cucumis* species, musk melon *(C. melo*) and cucumber (*C. sativus*). Sex expression in these two species is regulated by highly orthologous and conserved genes related to ethylene biosynthesis and signalling pathways (Boualem et al., 2008). For instance, *CsACS2* and *CmACS7* correspond to the *m* locus governing andromonoecy in cucumber and melon respectively. Mutation in M locus leads to bisexual flowers (Boualem et al., 2008; Boualem et al., 2009; Li et al., 2009). Mutations in *CsACS11* or *CmACS11* gene, which correspond to *a* (*a*ndroecy) loci, produce male flowers and androecious plants (Boualem et al., 2015), while similar androecious plants in cucumber are produced with mutation of *CsACO2* (Chen et al., 2016). So, it is clearly evident that in *Cucumis*, ethylene plays a major role in sex determination. However, mechanisms associated with sex regulation in *Momordica* is still unknown.

Bitter gourd is primarily a monoecious species; however, gynoecious lines with complete femaleness are reported from China, Japan, and India (Ram et al., 2002; Behera et al., 2006; Iwamoto and Ishida, 2006). Gynoecy has potential applications in heterosis breeding in exploiting earliness, high yield, quality and resistance through hybrid development. Use of gynoecious line as female counterpart has substantially decreased the hybrid seed cost and enhanced genetic purity of hybrids (Dey et al., 2010).

Gyneoecious phenotype in cucurbits arises either due to stamen arrest by enhanced ethylene production (Trebitsh et al., 1997; Mibus and Tatlioglu, 2004; Boualem et al., 2009) or anther specific DNA damage (Gu et al., 2011). Both recessive and dominant gene control of gynoecy is reported in cucurbits; in cucumber gynoecy is controlled by dominant gene named *Acr/stF/AcrF/F* (Shifriss, 1961; Galun, 1962; Kubicki, 1969; Robinson et al., 1976). The *F* locus encodes a duplicated copy of *CsACS 1* gene named as *CsACS1G* (Mibus and Tatlioglu, 2004). So, gynoecious line with additional ethylene synthase gene (*CsACS1G*) produces more ethylene leading to complete femaleness (Atsmon and Tabbak, 1979). On the other hand, Gynoecious sex expression is controlled by single recessive gene in muskmelon (*g)* and watermelon *(gy)* (Poole and Grimball, 1939). Gynoecy in water and musk melon is due to mutation in male determinant *CmWIP/CmWIP1 locus*, which encodes a *C2H2* zinc-finger-type transcription factor accountable for carpel abortion in bisexual flowers (Martin et al., 2009; Zhang et al., 2020). The genetic of gynoecy is well documented in bitter gourd and is known to be controlled a single recessive gene (Behera et al., 2006; Ram et al., 2006; Behera et al., 2009; Matsumura et al., 2014) which is similar to other species is controlled by the WIP gene. First genetic mapping of gynoecy locus was done by Matsumura et al. (2014) to a 12.73-cM genetic interval. Later on Cui et al. (2018), also mapped the gy locus to a 3.5-Mb physical interval between 17,619,724 bp and 21,144,642 bp on MC01.

Recently Fine mapping of gynoecy locus and candidate gene detection was done by Cui et al., 2022 by combining BSA seq and traditional molecular marker linkage analysis. Study mapped gynoecy locus i. e Mcgy1 locus into a 292.70-kb physical interval of 20,851,441 - 21,148,382 bp on MC01. Homologous gene of WIP or CsACS1G was not found Mcgy1 locus, signifying that the casual candidate of Gynoecy sex may be due to a novel gene than the previously reported in other cucurbits. Based on evaluation of candidate mutations in the gynoecy locus and gene expression studies and gene annotation information (Cui et al., 2020), gene MC01g1681 encoding Cytidine triphosphate synthase (CTPS), was considered as the candidate gene of Mcgy1. CTPS plays rate-limiting role in final step of *de novo* synthesis of cytidine triphosphate (CTP), which is essential for DNA, RNA and phospholipid biosynthesis in all organisms (Daumann et al., 2018). In the same study, RNA seq analysis identified several other candidates genes for gynoecy including, MC02g0607 belonging to AGL1 MADS-box family; MC05g0014 is an uclacyanin3-like gene; and MC04g1310. The identified novel candidate genes doesn’t seem to have any direct connection with ethylene biosynthesis/signaling. Thus, a different mechanism might be controlling gynoecy sex expression in bitter gourd. However, several studies have indicated the role of phytohormone perticluarly ethylene in sex expression of bitter gourd. Cui et al., 2022 observed differential expression of ethylene signal transduction genes, MC11g0603, encoding a Constitutive Triple Response 1 (CTR1), and MC04g0109, encoding an Ethylene Insensitive 3 (EIN3), between monoecious and gynoecious lines. CTR1 is known to promote biosynthesis of ethylene (Leclercq et al., 2002), whereas EIN has the inhibitory effect on ethylene biosynthesis (Wang et al., 2020). Use of ethylene inhibitory agents such as, Silver nitrate treatment for altering the sex forms in *Momordica* is reported (Hossain et al., 1996). *In-Silico* gene expression analysis by Gunnaiah et al., 2014 also reported ethylene biosynthesis and regulation genes as putative candidates for gynoecy expression in bitter gourd. These results suggests that ethylene play an important role in the formation of gynoecy in bitter gourd. Cui et al., 2022 also identified several other genes involved in the in plant hormone signal transduction, such as that of gibberellin and auxin. Hence, similar to other cucurbits, phytohormone and their cross talk may be associated with formation of gynoecy in bitter gourd. More elaborated studies need to be conducted to narrow down to exact candidate genes and to identify the molecular mechanism regulating gynoecy in bitter gourd.

Cross-talk among the important phytohormone is the well-known pathway associated with sex differentiation in cucurbits (Mandal et al., 2022). In addition to ethylene, other plant hormones like auxin, brassinosteroids (BRs), gibberillic acid (GA) and ABA also contribute to sex determination either by influencing ethylene biosynthesis and signalling or through ethylene independent pathways (Rudich et al., 1972; Trebitsh et al., 1987; Yin and Quinn, 1995; Papadopoulou and Grumet, 2005; Zhang et al., 2014; Tao et al., 2018). Exogenous auxin has feminizing effect in cucumber sex expression (Rudich et al., 1972; Trebitsh et al., 1987) and *C. maxima* (Wang et al., 2019) through up regulation of ethylene biosynthesis and signalling genes. Similarly, Gibberellic acid (GA) also exhibit male promoting effect on monoecious cucumber (Peterson and Anhder, 1960), musk melon and watermelon (Girek et al., 2013; Zhang et al., 2017). The masculinizing effect of GA is either through inhibition of ethylene biosynthesis (Yin and Quinn, 1995) or ethylene-independent manner (Zhang et al., 2014; Zhang et al., 2017). Besides, ABA is known to promote maleness in cucurbits through inhibition of ethylene biosynthesis by down-regulating of ACO genes (Gao et al., 2015; Lee et al., 2017). However, BRs (brassinosteroids) are known to promote ethylene induced femaleness thus, indirectly participate in cucumber sex determination (Papadopoulou and Grumet, 2005; Pawełkowicz et al., 2012). Similar to BA, cytokinin also acts as regulatory switch to increase the level of the ethylene by enhancing the stability of the ACS proteins (Lee et al., 2017). In brief, ethylene is considered as key regulator in sex determination of cucurbits and influence of other plant hormones in sex expression mainly through cross-talk with ethylene.

Marker assisted breeding has significantly accelerated the crop improvement programme. Success of Marker Assisted Selection (MAS) depends on QTL analysis though construction of high-density linkage map and identification of reliable and tightly linked marker to the trait of interest. However, application of molecular breeding in bitter gourd is limited due to the unavailability of decisive linkage map and scarcity of polymorphic markers (Rao et al, 2018). Therefore, the molecular basis of many economic traits is still unknown, and the use of molecular breeding in bitter gourd improvement programmes is still in its infancy. Advancement in sequencing chemistries and availability of next generation sequencing (NGS) techniques provide cheaper, rapid and efficient methods for high-density SNP discovery and genotyping in large populations (Davey et al., 2011). The availability of whole genome sequence of bitter gourd in public domain has served as an ideal resource for genome-wide identification of SSR and SNP markers *in silico* (Urasaki et al., 2017; Cui et al., 2020; Matsumura et al., 2020). This has encouraged researchers to work on genetic map construction, fine mapping and MAS of bitter gourd (Cui et al., 2018; Rao et al., 2018; Rao et al., 2021; Kaur et al., 2022). Recently, QTL-seq has been used as efficient tool for rapid mapping of QTLs and identification of candidate genes in less time and cost (Takagi et al., 2013). It has been successfully employed in mapping of economic traits in variety of vegetable crops including cucumber and tomato (Lu et al., 2014; Illa-Berenguer et al., 2015; Ruangrak et al., 2018). Hence, in the present study QTL-Seq analysis was performed to identify the candidate gene/s associated gynoecious sex expression in PVGy -201. Although sex expression is a vital developmental process in plant sexual reproduction, it is poorly reported in bitter gourd. Elucidating the mechanism underlying flower development and sex expression serves as a valuable resource for sex manipulation for academic and economic benefits in bitter gourd and related crops.

## Materials and methods

### Plant materials and phenotyping

**Figure 1:**
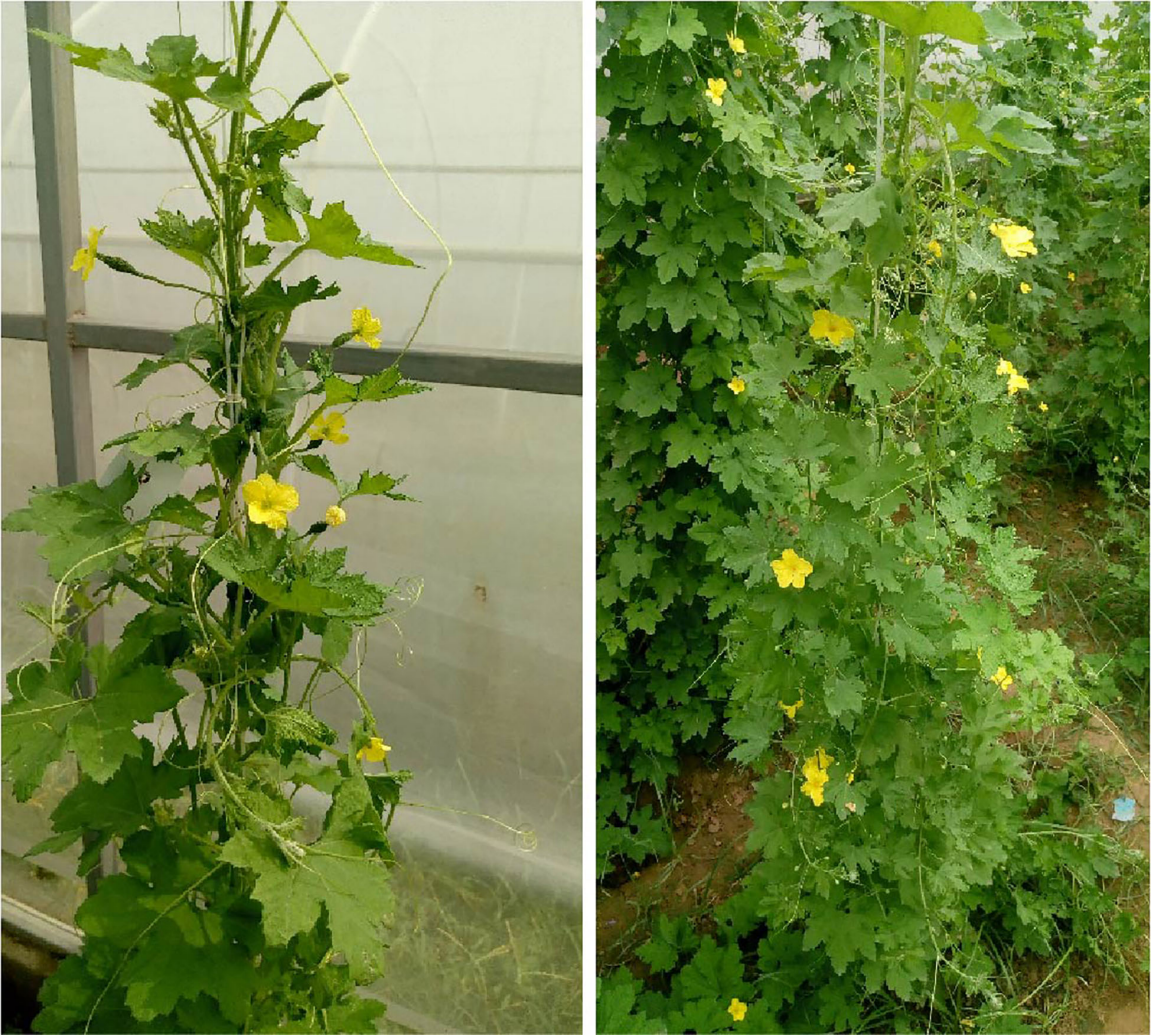
The parents with contrasting behaviour for sex expression **(A)** gynoecious line, PVGy-201 with only female flower **(B)** Monoecious line, Pusa Do Mousami with < 5% female flowers in any plant.

Two parental lines (PVGy-201 and Pusa Do Mausami) showing contrasting sex expression patterns were chosen as parents. The line PVGy-201 is a gynoeious line developed by transferring gynoecy trait to the Pusa Vishesh background. It exhibit stable-complete gynoecy (100% femaleness) and high female flower production even in temperature at high as 38-40°C. Furthermore, it is easy to maintain through male inducing chemicals such as silver nitrate and silver thiosulphate. The male parent, monoecious line Pusa Do Mausami (PDM), exhibits high male expression (> 95%) and delayed female appearance, hence serves as ideal monoecious counterpart. Since androecous lines with complete maleness are not yet reported in bitter gourd (Kole et al., 2020), monoecious lines with high male tendency such as PDM is used for genetic studies. (**Figure 1**; **Table 1**). The F_1_ plants were generated by the cross PVGy-201 × Pusa Do Mausami during Kharif, 2018 and F_2_ generation was obtained by self-fertilization (pollination of female flowers with pollen from the same plant) of the F_1_ plants during the spring-summer, 2019. The final experiment with 147 F_2_ plants along with parents were grown during Kharif season of 2019. The aforementioned population generation and phenotyping work was conducted at the Vegetable Research Farm of Indian Agricultural Research Institute (IARI), New Delhi, India.

**Table 1:** Flowering related traits of parents, PVGy-201 and PDM used in the present study.

Traits	PVGy-201	PDM
Node to first male flower	–	7.8
Node to first female flower	5.7	13.7
Days to first male flower	–	34.9
Days to first female flower	37.6	54.5
Percentage maleness	0%	>95%
Percentage femaleness	100%	< 5%

Sex expression of each plant was determined by recording the sex form of every flower produced from each plant. Subsequently using this data, male to female sex ratio of each F_2_ plant was computed. The plant with 100% female flowers was considered as gynoecious and those with both male and female flowers are considered as monoecious. The gynoecious plants with highest female flowers were used to formulate gynoecious bulk (G-bulk) and those with higher male flower percentage (>95%) were included in monoecious bulk (M-bulk). Each bulk contained 12 F_2_ plants exhibiting contrasting sex expression were taken for QTL-seq analysis.

### DNA extraction and whole genome re-sequencing of parents and bulked DNA

Total genomic DNA was extracted from young-fresh leaves of the parents (PVGy-201 and PDM), G- bulk and M-bulk (Doyle and Doyle, 1987). DNA concentration was determined using Nano Drop 8000 (Thermo Fisher Scientific, Waltham, MA), and equal amounts (1000 ng) from each of the 12 individuals constituting a bulk were pooled. Library construction and whole genome re-sequencing of the parents and the two bulks was performed as previously described (Abe et al., 2012; Takagi et al., 2013). Sequencing libraries with 250-600 bp insert sizes were prepared. Paired-end sequence reads (2 × 150 bp) of each library were obtained by Illumina HiSeq X. Adapter and low-quality sequences (<Q20) were trimmed by using fastp program (Chen et al., 2018).

### QTL-seq analysis

Sequencing data of parental lines and pooled F_2_ individuals were applied to QTL-seq pipeline developed by Takagi et al. (2013) for identifying location of gynoecious QTL. Briefly, in the pipeline, clean reads of PDM (monoecious parent) were aligned to reference genome sequence of OHB3-1 (Matsumura et al., 2020), and SNPs were called. By replacing these SNPs, PDM genome sequence file was developed. Thereafter, PVGy-201 (gynoecious parent) reads were mapped against the PDM genome sequence and SNPs between parental lines were defined. Subsequently, short reads of G-bulk and M-bulk were similarly aligned to PDM genome sequence. For each identified SNP locus between parental lines, SNP index was calculated as an allele frequency based on the sequence reads showing maternal or paternal allele. The SNP index is conferred as 0 if the entire short reads contain the PDM allele, while the SNP-index is 1 if all the short reads represent the PVGy-201-type allele. For clarifying SNP loci linked to gynnoecy, Δ (SNP-index) was then calculated in each locus by subtracting the SNP-index values between M-bulk and G-bulk, and sliding-window (10kb window size) of Δ (SNP-index) values were plotted for visualizing QTL region in the genome.

### Annotation of variants in the QTL region

For annotation of variants located in the candidate QTL region, PVGy-201-unique variants were extracted by reference mapping of sequence reads of both parental lines and applied to SnpEff software (version 5.0e, (Cingolani et al., 2012) with previously predicted gene models of reference genome (Matsumura et al., 2020).

### Promoter sequence variation analysis of putative candidate genes

To analyses the sequence variation, the sequence of the 12 putative candidate genes from the reference genome OHB3-1 (Matsumura et al., 2020) were extracted from NCBI and the promoter regions were identified through extracting flanking sequence regions for all the desired genes (if gene is forward strand, we take –60 for 5’upstream and +10 for 3’downstream and -10 for upstream and +60 3’ downstream for reverse strand gene. The corresponding genes were identified from the both parental assemblies, PDM and PVGy-201 through Blast (https://blast.ncbi.nlm.nih.gov) and the promoter regions of all the genes in both parental assemblies were extracted and analysed for any sequence variation in the parental lines.

## Results

### Inheritance of the gynoecious sex expression

To determine the inheritance pattern of gynoecy, two parental lines PVGy-201 and PDM exhibiting contrasting sex expression, its F_1_ and F_2_ population were phenotyped for sex expression. PVGy-201 is a gynoecious line produce only female flowers while PDM is monoeious line bearing both male and female flowers separately with predominant maleness (> 95% male flowers). The F_1_ plants expressed monoecious phenotype and in F_2_, out of 147 plants, 104 were monoecious and 43 plants were gynoecious which was fit to a segregation ratio of 3:1. The phenotypic expression in F_1_ indicates the recessive nature of gynoecy and segregation pattern in F_2_ suggest monogenic recessive inheritance of gynoecy in the genotype, PVGy-201.

### Whole genome re-sequencing and mapping of reads

Genomic DNA of two parental line (PVGY-201 and PDM) and two extreme bulks (G- bulk and M- bulk) were subjected to whole genome re-sequencing. Two extreme bulks were prepared based on the phenotype data of F_2_ population. G-bulk and M-bulk each contained 12 plants with high female and male flower production, respectively. Illumina high-throughput sequencing generated 70.4 million and 154.08 million paired- end short reads (150 bp x 2) from PVGy-201 and PDM, respectively (**Table 2**). For two extreme bulks, 100.48 and 129.20 million short reads for G-bulk and M-bulk, respectively, from F_2_ population were obtained. Quality filtering of these reads was carried out and 97-98% of clean reads were employed for further analysis.

**Table 2:** Summary of whole genome re-sequencing data used for QTL-seq analysis in the present study.

Genotype	Number of plants for pooling	Sequence data obtained (GB)	Number of raw reads	Number of clean reads (%^a^)
**PVGy-201**	**-**	10.56	70,445,450	68,707,610 (97.5)
**PDM**	**-**	23.11	154,084,080	151,581,250 (98.4)
**G-bulk**	**12**	15.07	100,480,892	98,278,696 (97.8)
**M-bulk**	**12**	19.38	129,201,904	126,867,286 (98.2)

a Percentage represents the ratio of number of filtered reads from number of raw reads by fastp program.

### QTL-seq analysis

The two parental lines (PVGy-201 and PDM) and two extreme pools, G-bulk and M-bulk from the F_2_ population were paired-end (150 bp) sequenced with an Illumina HiSeq platform. In total 10.56, 23.11, 15.07 and 19.38 Gb of clean reads from PVGy-201 (30× depth coverage), PDM (67.97× depth coverage), G-bulk (44× depth coverage), and M-bulk (57× depth coverage) were generated, respectively. These short reads were aligned to the “OHB3-1” reference genome for SNP calling.

**Figure 2:**
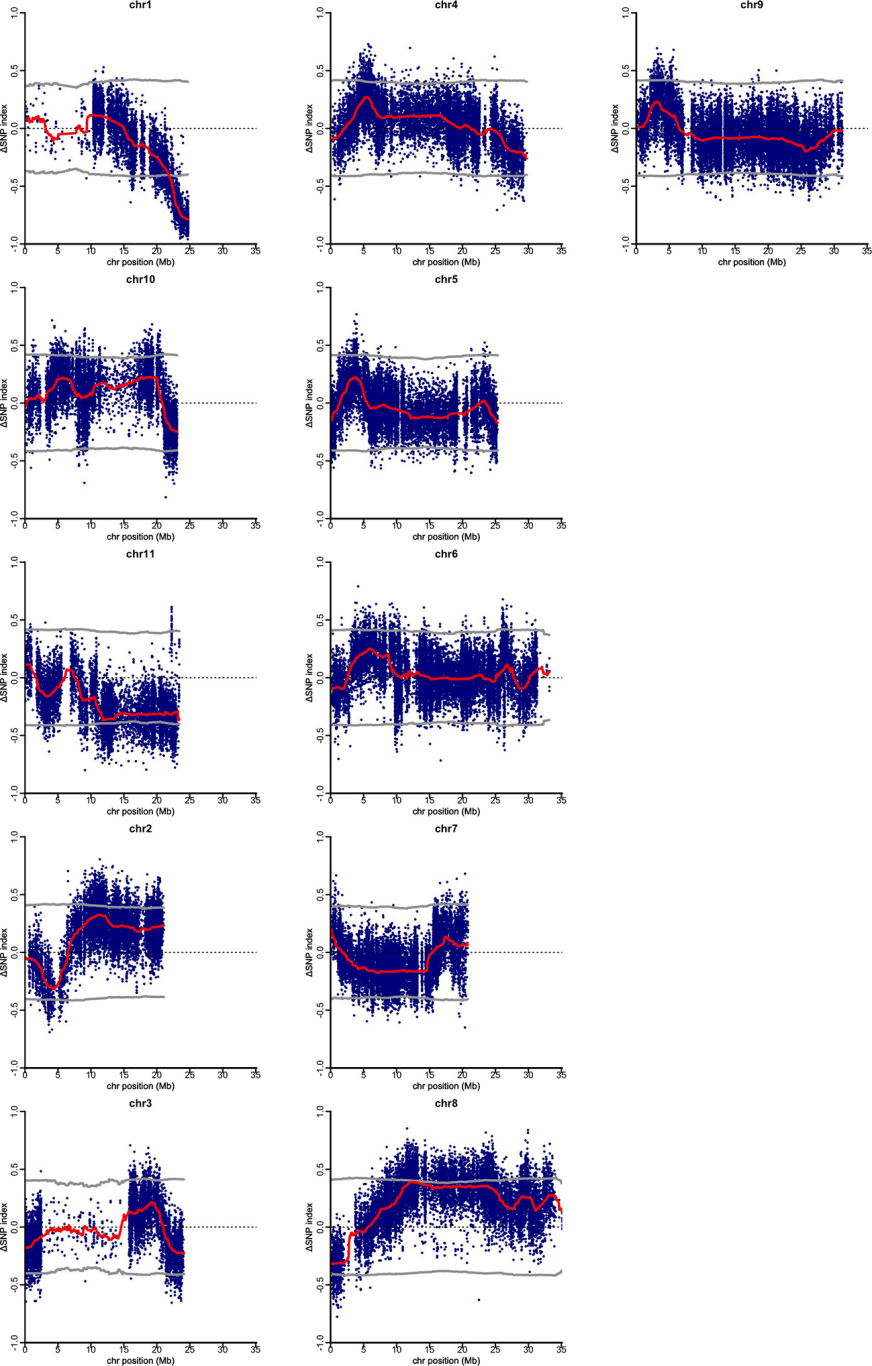
Quantitative trait loci (QTL)-seq identifies significant QTL on chromosome 1, for gynious sex expression in bitter gourd. ΔSNP (M-Bulk SNP Index–G-Bulk SNP Index) plotted against the physical position based *Momordica charantia* 11 chromosomes. The dark red line represents a sliding window of 2 Mb moving 500 kb intervals.

Using clean sequence reads, QTL-seq analysis for gynoecy was carried out. As the reference genome, pseudomolecule of OHB3-1 genome sequence was employed and 66,661 SNP loci were defined as homozygous qualified SNPs as markers. In the QTL-seq, allele frequency in each bulk sample at each SNP locus was calculated as the SNP-index. Also, for avoiding false positive signals, ΔSNP-index was employed as the difference of SNP-index between two bulk samples. In the present analysis, neutral SNPs in gynoecy were expected to show ΔSNP-index between 0.5 and -0.5 (**Supplementary Table 1**). Its value of the SNPs linked to gynoecious QTL should distribute around 1 or -1. According to sliding window plots of ΔSNP-index in each chromosome, only the region between 23.46 Mb and 24.7Mb on chromosome 1 exhibited significant unequal contributions (**Figure 2**). In QTL-Seq analysis the DNA samples of progenies of mapping population showing extreme phenotypic values are bulked and subjected to whole genome re-sequencing. We expect the bulked DNA to contain genomes from both parents in a 1:1 ratio for the majority of genomic regions. However, unequal representation of the genomes from the two parents is observed in the genomic regions harboring QTL for the phenotypic difference between “gynoecy” and “monoecy” bulks (Takagi et al., 2013). Thus, the G-bulk mainly had PVGy1-type genomic segments in the 23.46 Mb and 24.7Mb region of chromosome 1, whereas M-bulk had PDM-type genome in the same region, indicating that there is a major QTL differentiating PVGy1 and PDM located at this genomic region. Therefore, 1.31 Mb region on the chromosome 1 is considered as the candidate QTL associated with gynoecy in bitter gourd.

### Variants annotation and identification of candidate gene in in gynoecious locus

In the 1.31 Mb genomic region in chromosome 1 identified through the QTL-seq, the possible candidate genes with variant responsible for gynoecious sex expression in bitter gourd was explored. Therefore, PVGy-201-specific sequence variants in this region were selected and their effects to the structure of encoded genes (proteins) were estimated as annotation of variants. According to reference mapping of PDM and PVGy-201 reads and variant calling, 293,467 PVGy-201 unique variants, including SNPs and Indels identified. By using snpEff program and predicted gene model of the reference genome, impact of variants to the encoded proteins was predicted. After excluding variants located in intronic region and causing synonymous change, non-synonymous variants with high effect (frame-shift or stop-gained) and moderate effects (missense) on genes were selected. In the identified QTL region, a total of 1019 homozygous variants were identified between PVGy1 and PDM genomes. A total of 71 non-synonymous variants (SNPs and Indels) were identified, out of which 11 variants (7 Indels, 4 SNPs) were classified as high impact variants with frame shift/stop gain effect. Among the remaining 61 moderate impact variants (3 Indels, 58 SNPs) majority were missense variants. Variant annotation identified that, 71 non-synonymous variants were located in/associated with 41 protein coding genes (**Supplementary Tables 1**, **2**).

### Functional annotation of candidate genes

**Figure 3:**
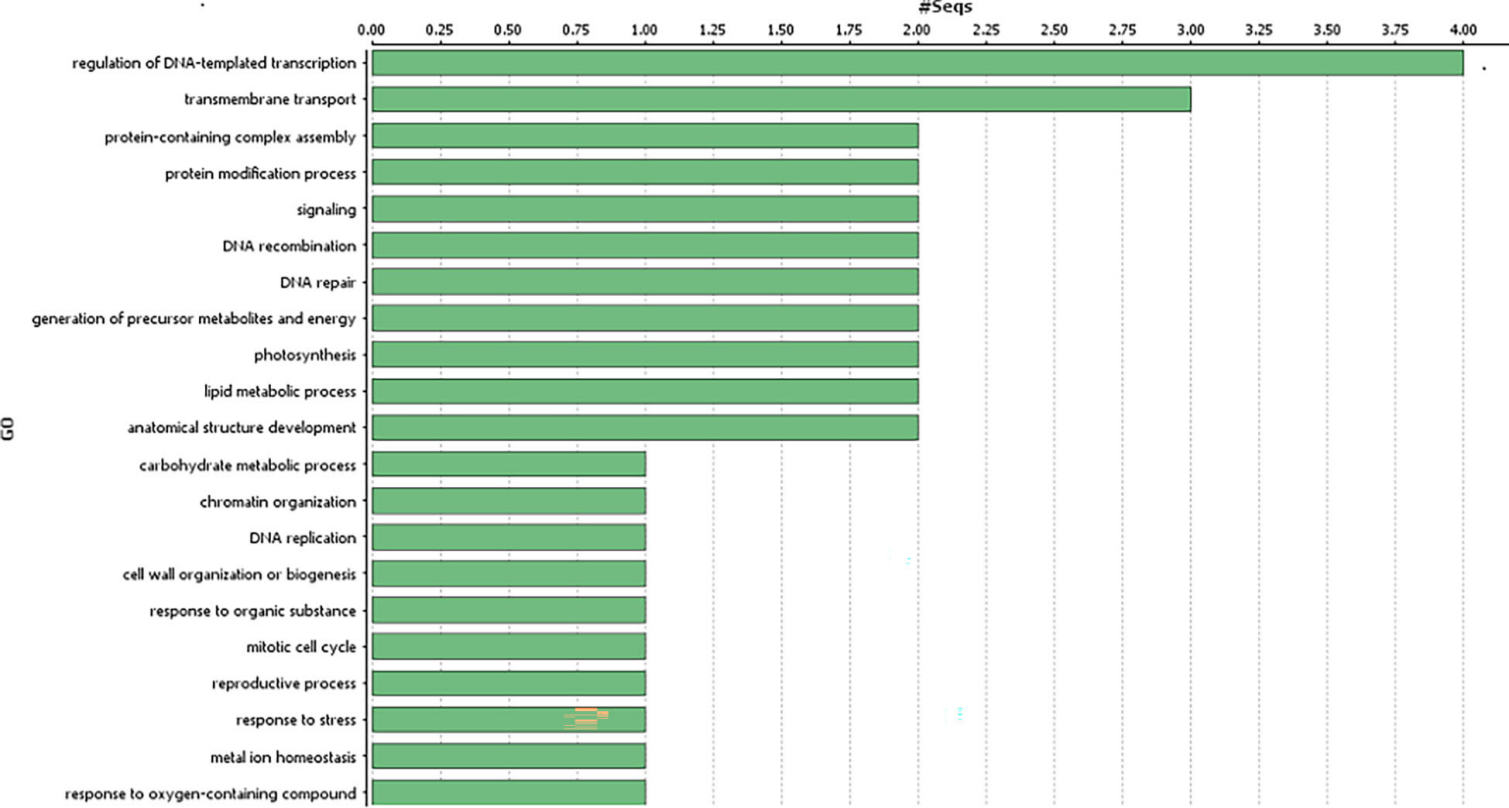
Gene ontology analysis of the identified genes in the genomic region associated with gynoecious sex expression in bitter gourd (Biological processes of the candidate genes associated with Non-synonymous variants).

**Figure 4:**
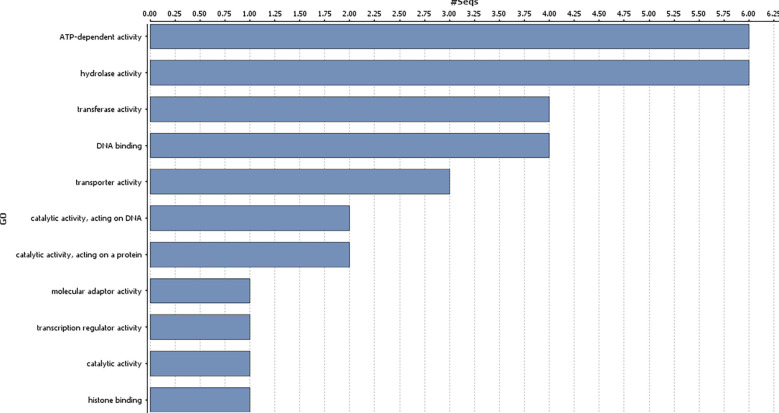
Gene ontology analysis of the identified genes in the genomic region associated with gynoecious sex expression in bitter gourd (Molecular functions of the candidate genes associated with Non-synonymous variants).

Genes harbouring these non-synonymous variants were annotated by BLASTx (Altschul et al., 1990) against the non-redundant protein database (http://www.uniprot.org/). Gene ontology classification revealed these 41 genes were mainly associated with biological processes (**Figure 3**), such as regulation of DNA-templated transcription, protein modification process, generation of precursor metabolites and energy, trans membrane transport, reproductive process, anatomical structure development; and molecular function, such as, transcription regulator activity, transporter activity, transferase activity, ATP-dependent activity etc. (**Figure 4**).

The biological processes of the 41 candidate genes were retrieved from the available bitter gourd genome data and Uniprot database. Among the 41 genes, 12 genes seemed to be related with flower development and sex expression, genes associated with male and female gametophyte development, male fertility restoration and phytohormone (auxin and ethylene) biosynthesis and signalling genes (**Supplementary Table 3**).

In the QTL region, 6 genes namely, Ethylene-responsive transcription factor 12 (Gene- LOC111025114), Copper-transporting ATPase RAN1(Gene-LOC111015725), Auxin response factor 6 (Gene-LOC111015731), CBL-interacting serine/threonine-protein kinase 23 (Gene- LOC111015768), LOB domain-containing protein 36-like (Gene- LOC111015703 and ABC transporter C family member 2 (Gene - LOC111015826) associated with development of gametophyte in association with important phytohomenes were identified (**Table 3**). Besides, another set of genes associated with male female gametophyte development and fertility restoration were identifies with possible role in determination of sex expression in bitter gourd. They were DEAD-box ATP-dependent RNA helicase 1 isoform X2 (Gene - LOC111015817), Polygalacturonase QRT3-like isoform X2 (Gene - LOC111015845), Protein CHROMATIN REMODELING 4 isoform X1 (Gene -LOC111015742), Pentatricopeptide repeat-containing protein (Gene - LOC111015813 and LOC111018538), putative F-box/LRR-repeat protein 23 (Gene -LOC111015857) and DNA replication licensing factor MCM6 isoform (Gene - LOC111015792).

**Table 3:** List of possible candidate gened associated with gynoecious sex expression in bitter gourd.

Sl no.	Candidate genes	Gene function
1	DEAD-box ATP-dependent RNA helicase 1 isoform X2	RNA metabolism (Li et al., 2011), male gametophyte (Li et al., 2011) and female gametophyte (Li et al., 2011) development
2	CBL-interacting serine/threonine-protein kinase 23	Sex differentiation by altering ethylene biosynthesis and brassinosteroid signaling (Pawełkowicz et al., 2012)
3	Pentatricopeptide repeat-containing protein At1g09900	Carpel development and male fertility restoration in CMS (Igarashi et al., 2016; Liu et al., 2016)
4	Auxin response factor 6	Feminizing effect in sex differentiation by promoting ethylene biosynthesis (Mibus & Tatlioglu, 2004; Liu et al., 2015; Niu et al., 2022)
5	LOB domain-containing protein 36-like	Pollen development (Xu et al., 2016), role in sex differentiation through altering brassinosteroid accumulation (Bell et al., 2012)
6	F-box/LRR-repeat protein 23	Pollen production and male fertility restoration (Gusti et al., 2009; Han et al., 2021)
7	ABC transporter C family member 2-like	Auxin transportation (Geisler et al., 2017; Rea, 2007), pollen development (Yadav et al., 2014; Chen et al., 2017)
8	Polygalacturonase QRT3-like isoform X2	Pollen wall development and pollen maturation (Rhee et al., 2003; Lyu et al., 2015), male fertility restoration (Shi et al., 2021).
9	Copper-transporting ATPase RAN1	Sex differentiation by activation of ethylene receptors in ethylene signal transduction pathway (Binder et al., 2010; Hirayama, N. et al., 1999)
10	Protein CHROMATIN REMODELING 4 isoform X1	Female gametophyte development (Huanca-Mamani et al., 2005) and stamen filament elongation (Zhao et al., 2021).
11	DNA replication licensing factor MCM6 isoform X1	Role in sex differentiation though alternating ethylene biosynthesis (Street et al., 2015; Wang et al., 2019)
12	Ethylene-responsive transcription factor 12-like	Sex differentiation by transcriptional regulation of ethylene biosynthesis genes (Zhang et al., 2009; Li et al., 2016).

### Promoter sequence variation analysis of putative candidate genes

The sequence variation in the promoter region of the 12 putative candidate genes located in the QTL region were analyzed. The four genes LOC111015768, LOC111015725, LOC111015857 and LOC111015742 did not exhibit any promoter region variation and rest eight putative candidates namely, ABC transporter C family member 2-like, Polygalacturonase QRT3-like isoform X2, DNA replication licensing factor MCM6 isoform X1, Auxin response factor 6, Pentatricopeptide repeat-containing protein, LOB domain-containing protein 36-like, Ethylene-responsive transcription factor 12-like, DEAD-box ATP-dependent RNA helicase 1 isoform X2 shown sequence variation in the promoter region (**Supplementary Table 4**).

## Discussion

Marker-assisted selection (MAS) is a powerful tool for accelerated breeding program that is quickly replacing tedious, expensive and time-consuming traditional phenotype-based breeding methods (Pandurangan et al., 2022) and applied widely in cucumber (Dey et al., 2020). QTL analysis through construction of high-density linkage map is a fundamental approach for molecular dissection of quantitative traits. Several multi-locus dominant DNA markers including RAPD (Dey et al., 2006; Paul et al., 2010), ISSR (Singh et al., 2007), and AFLP (Gaikwad et al., 2008) have been reported for genetic study of bitter gourd. However, in bitter gourd there is scarcity of genetic markers in public domain for the construction of a genetic map and marker-assisted selection (Tang et al., 2007).

Swift advancement in high-throughput sequencing methods and bioinformatics tools made detection of genome wide genetic polymorphism precise, quick and cheaper (Salvi and Tuberosa, 2005). Therefore, rapid identification of candidate genomic regions associated with a trait of interest through the “QTL-seq” method has been successfully applied in number of crops (Lu et al., 2014; Ruangrak et al., 2018; Shrestha et al., 2021). It combines advantages of bulk segregant analysis (BSA) and whole-genome re-sequencing for efficient genetic mapping (Takagi et al., 2013). Recently, QTL-seq is used widely and preferred over other traditional QTL mapping strategies. It is applied for genetic analysis of various economic traits in cucurbits such as early flowering (Lu et al., 2014), fruit length (Wei et al., 2016), subgynoecy (Win et al., 2019) in cucumber, heat tolerance in bottle gourd (Song et al., 2020) and mosaic resistance in zucchini (Shrestha et al., 2021). However, till now there is no study focusing on the successful application of QTL-seq for trait discovery in bitter gourd is reported.

### Genetic analysis of gynoecious trait in bitter gourd genotype, PVGy-201

In bitter gourd, F_1_ hybrids are preferred due to earliness, high yield, quality and tolerance to biotic and abiotic stresses (Behera et al., 2009; Dey et al., 2012). However, due to predominance of monoecy sex condition in bitter gourd, manual bagging and hand pollination is practiced for hybrid seed production which is costly and labour-intensive. On the other hand, use of gynoecious lines that produces only female flower as female parent can economise hybrid seed production with increased seed yield and hybrid genetic purity (Dey et al., 2010). Identification of gene(s) controlling gynoecism and (or) tightly linked markers would ease the identification gynoecious lines and hence their utilization in breeding programme. Furthermore, tightly linked marker can help in quick transfer of the gynoecious trait to the desirable parental background. Most genetic studies have reported the single recessive gene (*gy-1*) control of gynoecism (Ram et al., 2006; Behera et al., 2009; Matsumura et al., 2014), whereas involvement of two pairs of genes was reported recently by Cui et al. (2018).

### Identification of genomic region associated with gynoecious trait through QTL-seq

In the current study, QTL- seq analysis through comparison of Δ SNP-index graph of G-bulk and M-bulk, 1.31 mb region on chromosome 1 (spanning between 23.46 Mb to 24.7Mb) was identified as the candidate genomic region associated with gynoecious sex expression in bitter gourd. Earlier, using RAD (restriction-associated DNA) based genetic maps in bitter gourd Matsumura et al. (2014) identified a SNP marker, GTFL-1 linked to the gynoecious locus at a distance of 5.46 cM. Later on, Cui et al. (2018) identified QTLs for gynoecy and female flower number using genotyping by sequencing of F_2:3_ population of bitter gourd. Recently, a total of 22 QTLs for four sex expression-related traits namely gynoecy, sex ratio, node and days at first female flower appearance were mapped on 20 Linkage groups (Rao et al., 2018). This study identified a gynoecious (*gy-1*) locus flanked by markers TP_54865 and TP_54890 on LG 12 at a distance of 3.04 cM to TP_54890. In the QTL region identified in present study total of 71 non-synonymous variants (high and moderate impact) associated with 41 protein-coding genes were found. Among the 41 genes, 12 genes seemed to be related to flower development and sex expression; genes associated with male and female gametophyte development, male fertility restoration and phytohormone (Auxin and Ethylene) signalling genes.

### Identification candidate genes associated with ethylene biosynthesis

Similar to other cucurbits, in bitter gourd also ethylene is known to play key role in gynoecious sex expression (Matsumura et al., 2014). Analysis of draft genome (monoecious inbred line, OHB3-1) sequence of bitter gourd revealed the presence of orthologous sequences of major ACC synthase genes in the genome. *MOMC3_649* in bitter gourd was presumed to be an ortholog of *CmACS11* (female flower determinant in melon) and two proteins, *MOMC46_189* and *MOMC518_1* were similar to *CmACS-7* that control unisexual flower development in melon (Urasaki et al., 2017). These findings suggest that the sex determination of *M. charantia* is similar to that of *Cucumis melo* and *Cucumis sativus*, which is under the control of ethylene biosynthesis pathways.

Ethylene is considered as a key regulator of sex expression across members of *Cucurbitcea* family (Yin and Quinn, 1995; Boualem et al., 2015). “One hormone hypothesis” explains the dual role of ethylene in deciding sexual morph of individual flower, inhibition of maleness and promotion of femaleness (Yin and Quinn, 1995). Ethylene biosynthesis involves series of enzymatic reactions involving activity of 1-aminocyclopropane-1-carboxylic acid (ACC) synthase (ACS) and 1-aminocyclopropane-1-carboxylic acid oxidase (ACO) (Adams and Yang, 1979; Yang and Hoffman, 1984). Locus *‘a’* in melon and *M* locus in cucumber are orthologs, encoding the rate-limiting enzyme in ethylene biosynthesis namely, *CmACS-*7 and *CsACS-2*, respectively. In cucumber, locus *A* encodes *CsACS11* a member of ACS gene family and gynoecy governing *F* locus, codes for a duplicated ACS gene *CsACS1G* (Trebitsh et al., 1997; Mibus and Tatlioglu, 2004). Interestingly, exogenous ethylene application is involved in up-regulation of ethylene biosynthesis genes *CsACS7/CmACS7 and CsACS11/CmACS11* in cucumber and melon, respectively (Switzenberg et al., 2014; Tao et al., 2018). To date, except for *WIP1* all the genes reported in cucumber and melon are involved in ethylene biosynthesis, this clearly establishes the significance of ethylene in sex expression. Furthermore, in cucumber, application of exogenous ethylene, ethylene releasing agent (ethephon), or the ethylene precursor ACC promotes the formation of female flowers in monoecious plants (Yamasaki et al., 2003), while interference with ethylene synthesis (with aminoethoxyvinyl-glycine; AVG) or signalling (with AgNO_3_) induces male flowers in gynoecious plants (Takahashi and Jaffe, 1984).

Ethylene-responsive transcription factor (ERFs) proteins transcriptionally regulate ethylene-responsive genes *via* interaction with *cis*-acting elements or DRE/CRT motif located in the promoter region (Zhang et al., 2009; Li et al., 2016). In cucumber, ethylene response factor, *CsERF110/CmERF110* and *CsERF31* are known to transcriptionally regulate ethylene biosynthesis genes by directly binding to promoters of *CsACS11*/*CmACS11* and *CsACS2*, respectively (Pan et al., 2018; Tao et al., 2018). These results provide compelling evidence that ERF proteins, which control the transcription of the ACS and ACO genes, play vital role in ethylene biosynthesis in plants. The ethylene receptors *ETR1* and *ETR2* also play an important role in sex determination of cucurbits. The ethylene-insensitive mutant’s *etr1a* and *etr2b* of *Cucurbita pepo* both disrupt female flower development (converting monoecy into andromonoecy) and significantly increase the number of male flowers in the plant. This probably indicates that *ETR1* and *ETR2* are able to integrate the two ethylene biosynthesis pathways, perceiving and signalling the ethylene produced by *ACS2/7* as well as that produced by *ACS11* and *ACO2* (García et al., 2020).

Copper-transporting ATPase RAN1 (Gene-LOC111015725) identified in the QTL-region is another critical gene associated with ethylene response. *RAN1* is reported to be essential factor for male flower development in fig and cucumber (Mori et al., 2017; Terefe, 2005). Further, Terefe (2005) indicated that the *CsRAN1* gene is probably linked to the determining A/a gene in cucumber. *RESPONSE TO ANTAGONIST1 (RAN1)*, which encodes copper-transporting *ATPase* enzyme crucial in the first step of ethylene perception (Woeste and Kieber, 2000). RAN1 transports copper ions from the cytoplasm to the Golgi apparatus and plays a vital role in the biogenesis and activation of the ethylene receptors, *ETR1* (ETHYLENE RESISTANCE 1), *ERS1* (ETHYLENE RESPONSE SENSOR 1), *ETR2*, *EIN4* (ETHYLENE INSENSITIVE 4), and *ERS2* in plants (Binder et al., 2010). In an active ethylene signal-transduction process, the expression of *ACS11* relieves the inhibitory effect of *WIP1* on *ACS2* (Martin et al., 2009; Hu et al., 2017). *ACS2* promotes ethylene synthesis through positive feedback, increases cellular ethylene levels which in turn promotes pistil formation and inhibits stamen development. A mutated *ran1* allows *WIP1* expression through reduced sensitivity of ethylene synthesized by *ACS11* or *ACS7*, leading to stamen formation or male flower induction.

In plants, auxin is known to enhance endogenous ethylene production by promoting the expression of *ACS* genes and *ERF* genes (Trebitsh et al., 1987; El-Sharkawy et al., 2014). For instance, in *Arabidopsis* exogenous auxin treatment can significantly increase the expression of *AtACS4* and induce more ethylene (Stepanova et al., 2007). In cucurbits, auxin along with ethylene plays vital role in flower development and sex determination (Rudich et al., 1972; Friedlander et al. (1977). Auxin exhibits feminizing effect, evident from transformation of male flower buds into female flower buds (Galun, 1962) and increased female flower rate in cucumber (Takahashi and Jaffe, 1984) upon exogenous IAA treatment. Exogenous IAA treatment resulted in up-regulation of ethylene biosynthesis-related genes, ACC synthases (*CsACS1*, *CsACS2* and *CsACS11*) and ACC oxidases (*CsACO1*, *CsACO3*, and *CsACO4*) involved in sex determination in cucumber (Niu et al., 2022). Furthermore, presence of potential auxin-responsive elements (AREs) in the promoter region of *CsACS1* and *CsACS1G* demonstrated the mutual cross-talk in between the auxin and ethylene in the development of female flowers in cucumber (Mibus and Tatlioglu, 2004; Knopf and Trebitsh, 2006). Hence, auxin possibly affects sex determination in cucurbits *via* ethylene promoting ethylene production by enhancing the expression of ethylene biosynthesis and signaling genes (Takahashi and Jaffe, 1984; Trebitsh et al., 1987). Auxin Response Factors (ARFs) are crucial for the growth of pistils in Japanese Apricot (Song et al., 2015) and several auxin response elements (*CpARFs*) are reported to be involved in increased expression of ethylene signalling (*CpETR*) and biosynthesis (*CpACS*, *CpACO*) genes (Liu et al., 2015). In recent study, in cucumber, exogenous IAA treatment increased the transcription of *ERF (CsESR2 and Csa4G630010)* genes and one auxin response factor, *ARF* gene (*Csa2G000030*), suggesting that they may play regulatory roles in this crosstalk (Niu et al., 2022). Two auxin response factors *CsARF13* and *CsARF17* act as upstream regulators of *Cucumber MADS-box 1* (*CUM1)* (Ran et al., 2018), AG homolog in cucumber which expresses specifically in the stamens and carpels (Perl-Treves et al., 1998). *AGAMOUS (AG)* is a MADS-box gene that determines stamen and carpel development in *Arabidopsis*.

Expression of plant MCMs (Mini-chromosome maintenance protein complex) is greatly spread during the entire cycle (Huang et al., 2003). The function of plant MCMs is to prevent extra rounds of DNA replication (Brasil et al., 2017). Gene expression analysis in gynoecious and weak gynoecious cucumber identified correlated expression of many cell cycle pathway genes and ethylene related genes (Wang et al., 2019). An ethylene biosynthesis gene gyneocy determinant gene *CsACS1* (*G*), two ERFs (*CsERF12* and *CsERF118*) and two Ethylene receptors, *CsETR1* and *CsETR2* exhibited consistent expression pattern with cell cycle pathway and the gene (*Cs-MCM6*, *Cs-MCM2*, *Cs-CDC45*, *Cs-CDC20*, and *Cs-Dpri*) involved in the, *CsACS1* (*G*). Interestingly, ethylene also known to regulate cell cycle to inhibit plant growth during environmental stress conditions (Street et al., 2015). Integrating the results of these studies, it seems that the cell cycle genes may be involved in sex differentiation of cucumber initiated by ethylene, so there is a regulation relationship between cell cycle genes (*Cs-MCM6*, *Cs-MCM2*, *Cs-CDC45*, *Cs-CDC20*,and *Cs-Dpri*) and ethylene related genes (*CsERFs*, *CsETRs*, *CsACS1(G)*, *CsACO1* and *CsACO3).*

### Identification of other important possible candidate genes

In *Cucumis melo*, dominant allele *Gy/gy* gene can be correlated with the putative serine/threonine kinase gene *CsPSTK1* (Pawełkowicz et al., 2012). Dominant *Gy* allele inhibits *CsPSTK1* gene which in turn negatively affects ethylene biosynthesis. On the other hand, when recessive *gy* is present, the inhibition is removed and the *CsPSTK1* gene has a positive effect on ethylene levels and femaleness is promoted (Pawełkowicz et al., 2012). A correlation exists between *BAK1*, a receptor in the brassinosteroids (BR) signalling pathway, and *CsPSTK1*, which suggests the involvement of *CsPSTK1* in BR signalling (Pawełkowicz et al., 2012). BR phytohormone indirectly take part in cucumber sex determination which increases the number of female flowers through promoting of ethylene production (Papadopoulou and Grumet, 2005; Wu et al., 2010). Auxin response factors (ARFs) and serine/threonine protein kinases were among the 54 genes linked to plant hormone signal transduction which shown differentially expression in male and female floral in the dioecious cucurbit ivy gourd *Coccinia grandis* (Mohanty et al., 2017).

In cucumber LOB protein (encoded by *Cucsa.098680*), contributes to pollen development, as reported by Xu et al. (2016). LOB protein negatively regulates the accumulation of brassinosteroids (BR) (Bell et al., 2012) a phytohormone which indirectly take part in cucumber sex determination, through promotion of ethylene biosynthesis (Papadopoulou and Grumet, 2005; Wu et al., 2010). The ABC transporter family genes are associated with trans-membrane transport of diverse substrates (e.g., lipids, heavy metal ions, sugars, amino acids, peptides, and secondary metabolites) and/or regulating other transporters (Jasinski et al., 2003; Rea, 2007). In *Arabidopsis* ABC transporters genes (*AtPGP1* and *AtPGP19*) are known to regulate auxin transportation (Rea, 2007). ABC transporters genes are also associated with pollen grain development in *Arabidopsis* (*AtABCG1* and *AtABCG16*) (Yadav et al., 2014) and pineapple (*AcABCG38)* (Chen et al., 2017). ABC transporters exhibited differential expression patterns in male, female, and hermaphroditic plants (Pawełkowicz et al., 2019).

RNA helicases are adenosine tri-phosphatases that unwind the secondary structures of RNAs and are required in almost every aspect of RNA metabolism (Liu et al., 2010). Programmed cell death (PCD) in tapetum degeneration is critical for development of male gametophytes in flowering plants. In rice, two putative DEAD-box ATP-dependent RNA helicases (encoded by *AIP1* and *AIP2*) are involved in tapetum degeneration during pollen development (Li et al., 2011). Furthermore, in *Arabidospis* DEAD/DEAH-box helicases were specifically enriched in the megaspore mother cell and a DEAD-box RNA helicase (encoded by *SWA3*) is known to be involved in female gametogenesis (Liu et al., 2010). These studies imply the role of DEAD/DEAH-box helicases in male and female gametogenesis. Polygalacturonase (PG) is a pectin-digesting enzyme involved in numerous plant developmental processes and is described to be of critical importance in pollen wall development. The *QRT3* gene encodes a divergent class of polygalacturonase (PG) that is transiently expressed in tapetal cells and reported to participate in the pollen maturation through tetrad pectin wall degradation and pollen wall formation (Rhee et al., 2003). *BoMF25* in *Brassica oleracea*, a homologous gene of *At4g35670* is known to encode PG that exhibits pollen specific expression and found to be essential for pollen wall development (Lyu et al., 2015). In *Arabidopsis thaliana* “*res2*” locus which encode QRT3, is associated with restoration of thermo/photoperiod-sensitive genic male sterility (P/TGMS) (Shi et al., 2021). Chromatin remodelling proteins are involved in various biological processes in eukaryotes. In Arabidopsis, chromatin remodelling proteins (CHR11 and CHR17) (Huanca-Mamani et al., 2005) and RINGLET proteins (RLT1 and RLT2) (Li et al., 2012) were identified as the members of the, Imitation of Switch (ISWI) complex. In *Arabidopsis ISWI* complex has role in female gametophyte development (Huanca-Mamani et al., 2005) and in stamen filament elongation by regulating Jasmonic acid (JA) biosynthesis (Zhao et al., 2021).

F-Box LRR is a large subfamily of the plant F-box family known to mediate target protein degradation in response to developmental and hormonal signals (Kuroda et al., 2002). In *Arabidopsis* an F-box/LRR-repeat protein similar to gene04153 is required for pollen mitosis II (Gusti et al., 2009). The homolog of this gene was found in the candidate QTL region associated with male sterility in straw berry *Fregeria vesca*. ssp. *bracteata* (Tennessen et al., 2013). In wheat an F-box/LRR-repeat protein (encoded by TraesCS1B01G085600) was known to be associated with male fertility restoration in TGMS line-YS3038 (Han et al., 2021). Earlier Mutation of gene encoding F-box/LRR (FBL) in *Arabidopsis* affected the fertility of the male gametes due to obstruction in transformation process of microspores from the uninucleate to the binucleate stages.

### Promoter sequence variation analysis of putative candidate genes

In cucurbits various sex forms are produced either due mutations in the coding region of the candidate genes and also due to mutation/variation in promoter regions of the candidate gene or even due to the copy number variation of candidate gene (Trebitsh et al., 1997; Mibus and Tatlioglu, 2004; Boualem et al., 2008; Zhang et al., 2015; Tao et al., 2018). In cucumber gynoecy is determined by the copy number variation (CNV)-based, dominant, and dosage-dependent *femaleness* (*F*) locus. Gynoecious plants contained three genes: *CsACS1, CsACS1G*, and *CsMYB*, of which *CsACS1G* is a duplication of *CsACS1* and loss of *CsACS1G* leads monoecy (Trebitsh et al., 1997; Mibus and Tatlioglu, 2004; Zhang et al., 2015). In melon a conserved mutation in the coding region CmACS-7 led to andromonoecious sex form (Boualem et al., 2008). Furthermore, Sex regulation in cucurbits is also due to transcriptional regulation of sex determining genes by various transcription factors that interacts with the with regulatory elements located in the promoters of ethylene biosynthesis and signaling genes (Zhang et al., 2009; Li et al., 2016). For instance in *cucumis* through a conserved mechanism, *CsERF110* and *CmERF110* respond to ethylene signaling, mediating ethylene-regulated transcription of *CsACS11* and *CmACS11* in cucumber and melon, respectively (Tao et al., 2018). These studies strongly indicates that the ethylene biosynthesis gene expressions are modified at transcriptional level by binding of regulatory proteins to promoter region of *ACS* and *ACO* genes. So, in bitter gourd also variation in promoter region might change the sex form. In the current study eight of the twelve putative candidate genes namely, ABC transporter C family member 2-like, Polygalacturonase QRT3-like isoform X2, DNA replication licensing factor MCM6 isoform X1, Auxin response factor 6, Pentatricopeptide repeat-containing protein, LOB domain-containing protein 36-like, Ethylene-responsive transcription factor 12-like, DEAD-box ATP-dependent RNA helicase 1 isoform X2 exhibited sequence variation in the promoter region. Therefore, genes are the most potential candidates determining gynoeciuous sex form in the bitter gourd genotype, DBGy-1. These findings suggests that the transcriptional regulation of sex determination genes play a major role in gynoecy expression in bitter gourd.

Large number of variants identified in the QTL-region will enable to develop molecular markers and fine mapping of the gynoecious sex expression in bitter gourd. The set of the possible candidate genes identified in the study with possible role in sex regulation will be instrumental for future study in bitter gourd and their functional analysis across different plant species.

## Conclusion

Gynoecious sex expression is an extremely important trait to facilitate economic hybrid seed production in cucurbits. The present study involving and F_2_ progenies of PVGy-201 × Pusa Do Mousami revealed that the gynoecious sex expression in the genotype, PVGy-201 is controlled by a single recessive gene. In the chromosome 1, 1.31 Mb regions was identified to be associated with gynoecious sex expression. A large number of variants were identified in the QTL-region which will be instrumental in fine mapping of gynoecious trait. Among the identified genes in the QTL-region, Ethylene-responsive transcription factor 12, Auxin response factor 6, Copper-transporting ATPase RAN1, CBL-interacting serine/threonine-protein kinase 23, ABC transporter C family member 2, DEAD-box ATP-dependent RNA helicase 1 isoform X2, Polygalacturonase QRT3-like isoform X2, Protein CHROMATIN REMODELING 4 were identified as possible candidate genes associated with gynoecious sex expression in bitter gourd because of their role in development of male and female gametophytes in number of crops. The findings in the study provides insight about sex expression in bitter gourd and will facilitate fine mapping and more precise identification of candidate genes through fine mapping and functional validation of the identified genes. The present study provides insight into the genetic and molecular basis of gynoecious sex expression in bitter gourd.

## Data availability statement

The data presented in the study are deposited in the NCBI repository, accession number PRJNA884851.

## Author contributions

Conceived theme of the study and designed experiment: TB. Data curation: HM, AS, SD, MI, SJ, TB. Investigation: VD, SD, KK, Boopalakrishnan G. Resources: SD, TB, VC, AM. Supervision: TB, AR, HM, SD, AM, RE. Visualization: TB, SD, AM, GJ. Writing original draft: VD, IP, TB, SD, SJ. Review and editing: TB, SD, SJ, MI, GJ. A All authors contributed to the article and approved the submitted version.

## Supplementary material

Supplementary Table 1 List of moderate and high effect variants (SNP/INDELs) located in QTL region associated with gynoecious sex expression in bitter gourd.

Supplementary Table 2 Functional annotation of candidate genes present in the identified QTL regions associated with gynoecious se expression in bitter gourd.

Supplementary Table 3 Effect of non- synonymous variants on candidate gene function/protein change.

Supplementary Table 4 Promoter sequence variation analysis of putative candidate genes.
